# Effects of flunixin meglumine on experimental tendon wound healing: A histopathological and mechanical study in rabbits

**Published:** 2013

**Authors:** Mehdi Behfar, Rahim Hobbenaghi, Farshid Sarrafzadeh-Rezaei

**Affiliations:** 1 *Department of Clinical Sciences, Faculty of Veterinary Medicine, Urmia University, Urmia, Iran;*; 2 *Department of Pathobiology, Faculty of Veterinary Medicine, Urmia University, Urmia, Iran.*

**Keywords:** Collagen, Inflammation, NSAIDs, Repair, Tensile strength

## Abstract

Tendons are frequently targets of injury in sports and work. Whether nonsteroidal anti-inflammatory drugs (NSAIDs) have beneficial effects on tendon healing is still a matter of debate. This study was conducted to evaluate effects of flunixin meglumine (FM) on tendon healing after experimentally induced acute trauma. Twenty eight adult male New Zealand White rabbits were subjected to complete transection of deep digital flexor tendons followed by suture placement. Treatment group received intramuscular injection of FM for three days, and controls received placebo. Subsequently, cast immobilization was continued for two weeks. Animals were sacrificed four weeks after surgery and tissue samples were taken. The histological evaluations revealed improved structural characteristics of neotendon formation including fibrillar linearity, fibrillar continuity and neovascularization in treatment group compared to those of controls (*p* < 0.05). However, no significant differences were found between two groups in terms of epitenon thickness (*p *> 0.05). Mechanical evaluation revealed significant increase in load-related material properties including ultimate load, yield load, energy absorption and ultimate stress in treatment group compared to those of control group (*p* < 0.05). However, no statistically significant differences in terms of stiffness and ultimate strain were found (*p* > 0.05). The present study showed that intramuscular injection of FM resulted in improved structural and mechanical properties of tendon repairs and it could be an effective treatment for acute tendon injuries like severance and laceration.

## Introduction

Tendons are frequently targets of injury in sports and work and significant dysfunction and disability can result from suboptimal healing of tendon injuries that exclude the patient from work and exercise for several months. ^1^

Nonsteroidal anti-inflammatory drugs (NSAIDs), also known as cyclooxygenase (COX) inhibitors, have been shown to influence wound healing in several tissues.^2^^-^^5^ These agents have been shown to diminish pain and inflammation and, therefore, improve the patient’s recovery and return to function.^6^ The prevailing argument is that healthy tissue is not inflamed. Therefore, if the inflammation in an injured tissue is stopped, the tissue remains healthy. The problem with this viewpoint is that, in addition to being a sign of injury, inflammation is a necessary component of the healing process. As noted by Leadbetter, inflammation can occur without healing, but healing cannot occur without inflammation.^7^ On the other hand, when the inflammatory process is prolonged, problems for the ligament or tendon undergoing healing are ensued. Continued inflammation leads to excessive adhesion formation, fibrosis, and scarring.^8^ Therefore, there is no clear consensus on the effect of NSAIDs on tendon healing.^9^ Experimental studies have shown variable results, both beneficial^10^^,^^11^ and mostly deleterious.^6^^,^^12^^-^^16^ Whether blockade of arachidonic pathways to limit inflammation has any effect on regeneration or healing processes in injured tendons is currently unknown.

Based on these observations, this study was designed to determine the effects of flunixin meglumine, a non-selective COX inhibitor, on histological and mechanical properties of the healed tendon in an experimentally induced tendon injury in rabbits.

## Materials and Methods

**Animals.** All protocols were reviewed and approved by the authors’ Institutional Research Council before animal experimentation. Twenty eight adult male New Zealand White rabbits weighing 2-2.5 kg, were studied. The rabbits were divided into two groups in a simple random method, with 14 rabbits in each group and assigned to the experimental protocol. They were allowed to acclimatize for one week before the start of the study. Animals were housed individually in cages with natural day/night cycle in an environment maintained at 21-23 ˚C. Rabbits had free access to commercial rodent food and water throughout the study period.

**Surgical procedure.** Rabbits were anaesthetized by intramuscular injection of 5 mg kg^-1^ xylazine hydrochloride (Alfasan, Woerden, The Netherlands) and 40 mg kg^-1^ ketamine hydrochloride (Alfasan, Woerden, The Netherlands).^17^ Then, one hind limb of each rabbit was randomly prepared for an aseptic surgical intervention. After skin incision, deep digital flexor tendon was exposed. The injury model was a sharp complete transection through the central one third of the tendon. Subsequently, tendon stumps were sutured with 3/0 monofilament nylon (Ethicon, Somerville, USA) in modified Kessler pattern. Then, the skin was sutured routinely. Cast coaptation and immobilization was continued for two weeks. No antibiotics were given to the model animals neither in control nor in treatment groups. Rabbits in treatment group were injected 2 mg kg^-1^ FM (Schering-Plough, Stockholm, Sweden) intramuscularly, once daily for three successive days and controls received saline solution as placebo. Four weeks after surgery, rabbits were euthanized with overdose of 50 mg kg^-1^ thiopental sodium (Biochemie, Kundl, Austria) and skin incision reopened. Tendons were harvested by proximal and distal transverse incisions 2 cm away from the repair site and processed for histological studies (n = 16). For mechanical evaluations, the harvested tendons (n = 12) were wrapped in saline soaked gauze and immediately stored at -20 ˚C.

**Histological analysis.** Longitudinal sections (5 μm in thickness) were stained with Hematoxylin and Eosin (H&E) and were evaluated under a light microscope (Nikon, Tokyo, Japan), equipped with an eyepiece graticule, for the following criteria: fibrillar linearity, fibrillar continuity, angiogenesis in neotendon, and epitenon thickness.^18^ Repaired areas including longitudinally oriented collagen fibers were histologically assessed. In this regard, total area of neotendon between the severed ends of tendons was measured at 40× magnification and the area consisting longitudinal pattern of collagen fibers was calculated. The ratio of the values was defined as the percentage of fibrillar linearity for each tendon. The width of the widest part of neotendon including fibers following the direction of those in native tendon was measured at 40× magnification in both tendon-neotendon junctions and the ratio of their mean to the mean width of junctions was defined as the percentage of fibrillar continuity for each specimen. To examine the rate of angiogenesis within the neotendon, the number of blood capillaries was counted at 100× magnification. For this evaluation, five randomly selected fields were examined per sample. The number of capillaries was averaged and reported for each specimen. The thickness of the epitenon was measured at 100× magnification in three points of epitenon (from the thickest to the thinnest parts of epitenon) on both sides of neotendon and the numbers were averaged for each specimen.

**Mechanical testing.** Prior to mechanical testing, suture materials were removed and tendons were allowed to thaw for 2 hours at room temperature. Tendons were submitted to a mechanical test of traction using H10KS (Hounsfield Ltd., Salfords, UK) testing machine. In order to prevent tendon slippage during tensile testing, 360 grit sandpaper was attached to the ends of each specimen for better clamping. The upper clamp was attached to a 500 N load cell and its displacement was controlled with the aid of a computer, endowed with QMat software (Version 2.22, Hounsfield Ltd., Salfords, UK), responsible for commanding the equipment and for plotting the force-elongation curve. The tendons were secured in the clamps and gauge length was defined as the length of the tendon under a 0.5 N pre-load. Under this load, width and thickness of the tendons were measured using a vernier caliper and the cross-sectional area (CSA) was calculated by assuming it to be elliptical. The dynamic testing was performed under axial tension with a constant speed of 50 mm min^-1^. The mechanical testing consisted of a single-cycle load-to-failure. The force and elongation of the tendon were continuously recorded until the flexor tendon failed. The mode of failure was visually observed and recorded. For each tendon the force-elongation curve was plotted and the following mechanical parameters were obtained: ultimate load (N), yield point (N), stiffness (N mm^-1^), maximum stress (N mm^-2^), maximum strain (%), and energy absorption (N mm).^19^ The ultimate load was defined as the maximum force measured in the tendon during the failure test. The yield point, defined as the point where the curve first deviated from the linear region. Energy absorption values were measured by calculating the area under the force-elongation curve up to the point of maximum force. Maximum stress obtained by dividing tendon maximal force by its CSA. Similarly, maximum strain was calculated by dividing the elongation at the point of maximum force by initial length and was expressed as percentage. Stiffness was determined as the maximum gradient in the linear region of the force-elongation curve.

**Statistical Analysis.** All data were analyzed by PASW Statistics (Release 18, SPSS Inc., Chicago, USA). Data were analyzed using Student's unpaired *t*-test and the level of significance was set at *p* < 0.05.

## Results

**Fig. 1:**
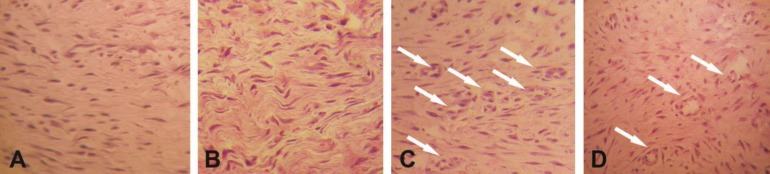
Photomicrographs showing the differences in the histological appearances between the groups. Fibrillar linearity in treatment group indicated superior remodeling of neotendon **(A)** compared to that of control group **(B)**. Arrows show the neovessels in neotendon which were significantly increased in treatment group **(C)** compared to those of control group **(D)**, (H&E, 100×).

In macroscopic gross evaluation of tendons, no evidence of faulty union and local or systemic complications was observed. Dehiscence of the suture with gap formation between the tendon stumps was not seen in any of the tendons. The results of histological evaluations are shown in Table 1. Four weeks after surgery, the site of repair was less cellular and began to show collagen fibers longitudinally oriented in treatment group. In contrast in controls, the granulation tissue was highly cellular with no properly oriented collagen fibers (Figs. 1A and 1B). 

**Table 1:** Results of histological evaluation of neotendons, four weeks after surgery (Mean ± SD).

**Groups**	**Fibrillar linearity (%)**	**Fibrillar continuity (%)**	**Number of capillaries **	**Epitenon thickness (µm)**
**Control**	18.24 ± 2.33	45.13 ± 3.44	26.48 ± 7.15	14.08 ± 3.91
**Treatment**	29.39 ± 4.58*	56.64 ± 6.23*	49.19 ± 10.22*	11.53 ± 2.31

* Asterisks indicate significant difference compared to control group in each column, (*p *< 0.05).

Histological studies showed that the differences between two groups in terms of collagen fiber linearity and continuity were statistically significant (*p* < 0.05, for both comparisons, respectively). Also, statistical analysis showed that the number of blood neovessels in the repair site was significantly higher in treatment group compared to controls (*p* < 0.05), (Figs. 1C and 1D). No significant differences were found between the two groups regarding epitenon thickness (*p* > 0.05).

In mechanical evaluations, failure mode was not influenced by treatment as there was rupture at the repair site in all tendons. Mechanical properties of tendons are shown in Table 2. Tensile strength parameters including ultimate and yield loads, energy absorption, and ultimate stress were significantly higher in treatment group compared to those of the control group (*p* < 0.05, for all comparisons). However, no significant differences were found in terms of stiffness and maximum strain between two groups (*p* > 0.05, for both comparisons).

**Table 2:** Mechanical properties of neotendons, four weeks after surgery (Mean ± SD).

**Groups**	**Ultimate load (N)**	**Yield load (N)**	**Energy absorption (N mm)**	**Stiffness (N mm** ^-1^ **)**	**Stress (N mm** ^-2^ **)**	**Strain (%)**
**Control**	5.27 ± 1.22	4.25 ± 1.32	9.21 ± 3.49	1.53 ± 0.36	1.75 ± 0.41	15.62 ± 2.54
**Treatment**	12.32 ± 2.50*	8.36 ± 3.05*	27.03 ± 6.88*	1.36 ± 0.20	4.02 ± 0.68*	16.35 ± 2.20

* Asterisks indicate significant difference compared to control group in each column, (*p* < 0.05).

## Discussion

The results of this study showed that successive injection of FM for three days could improve tendon repair in rabbits. In the present study, uniform neotendon formation indicative of improved fibrillar linearity and continuity in treatment group showed positive effects of FM on tendon healing. Longitudinal alignment of collagen fibers provides high tensile strength to withstand the large forces.^20^ It is believed that optimal healing occurs when the continuity of collagen fibers is maintained; otherwise, the mechanical properties of newly formed tissue would be inferior to those observed pre-injury.^21^^,^^22^ On the other hand, to store and release high loads without damage, tendons require a great energy-absorbing capacity. Thus, increasing the energy absorption capacity must be one of the main points in treatment of tendon injuries.^23^ The results of mechanical testing in the present study demonstrated significant increase in ultimate and yield loads and energy absorption in treatment group compared to those of the control animals. The improved repairs in mechanical properties of the present study, was in agreement with previous studies using aspirin, phenylbutazone and indomethacin in rat model.^24^^,^^25^

Tendon repairs in treatment group developed significantly greater ultimate stress values in comparison with that of control animals. It is believed that stress reflects the resulting matrix ultrastructure and biochemistry of tissues.^26^ Therefore, it seems an organized neotendon has been developed in response to treatment with FM as the histological studies showed a significantly improved fibrillar linearity in FM-treated group.

Tensile strength depends on the collagen fibril deposition^27^ and, fibroblast proliferation is essential for collagen synthesis.^28^ The significant higher tensile strength of repairs in treatment group indirectly demonstrates significant increase in collagen deposition due to fibroblastic hyperplasia. In an in vitro study, diclofenac and aceclofenac had no significant effect on tendon cell proliferation, however, indomethacin and naproxen inhibited cell proliferation within patellar tendons of rabbits.^29^ Another study showed that NSAIDs inhibited formation and maturation of collagen at tendon in a rat model.^30^

The results of the present study revealed that FM not only increased the formation of collagen, but facilitated linear orientation of collagen fibers. Linear orientation is a crucial step in tendon healing.^31^ This finding was consistant with improved organization of extracellular collagenous matrix that was resulted from flurbiprofen administration following Achilles tendon transection in rats.^32^ In view of the fact that the initial deposition of collagen produces relatively weak fibrils with random orientation and by maturity, the collagen fibers become more obviously oriented in line with local stresses.^33^ Apparently, FM might accelerate neotendon maturation which usually takes a long period in normal conditions.^34^

Another important aspect of healing is neo-vascularization and when it comes to wound healing, it is crucial to have enough angiogenesis, because without the restoration of blood flow, oxygen and nutrients cannot be delivered to the healing site.^35^ It has been reported that both selective (e.g., celecoxib, meloxicam and carprofen) and nonselective (e.g., diclofenac, naproxen and ibuprofen) NSAIDs inhibit angiogenesis through direct effects on endothelial cells.^35^ According to the results of this study, administration of FM provoked a better neovascularization within neotendon. We believe that reduced inflammation and degenerative process during the first phase of tendon healing have improved angiogenesis in response to FM administration. This consequently resulted in optimal healing through bridging and mechanical integrity of tendon repairs in treatment group.

Tendon repair is often complicated by formation of peritendinous adhesions resulting in loss of normal tendon gliding, ensuing limb or digital stiffness and functional disability.^36^ It is believed that the more epitenon thickness occurs, the more adhesion may occur between tendon and surrounding tissues^37^ which restrict movement of tendon after healing. Decreased epitenon thickness observed in treatment group, although statistically insignificant compared to controls, demonstrated that FM could inhibit abundant adhesion formation between the neotendon and surrounding soft tissues. Reportedly, ibuprofen, a non-selective COX inhibitor, resulted in reduced adhesion formation after healing of rabbit flexor tendon injury.^38^ Previously, Nishimura *et al.* demonstrated prevention of experimentally induced postoperative adhesions using ibuprofen, a nonselective COX inhibitor.^39^^,^^40^

Non-significant difference in tendon viscoelastic properties (i.e., stiffness and strain) in the present study between two groups could be due to low quantity of collagen cross-links.^41^^,^^42^ Reportedly, the final maturation stage occurs after ten weeks and during this period there is an increase in cross-linking of the collagen fibrils which causes the tissue to become stiffer.^20^ Therefore, at the time of sampling (four weeks after surgery), collagen fibers in all repaired tendons did not reach the time required for proper cross-linking.

In conclusion, the histological findings and mechanical functionality of neotendons in the present study suggested that a short-term intramuscular injection of FM could have beneficial effects on healing of acute tendon injuries like severance and laceration.
